# Significance of headache in intracranial vertebrobasilar artery dissections: an observational study

**DOI:** 10.1038/s41598-023-48941-5

**Published:** 2023-12-08

**Authors:** Seong-Joon Lee, Jin Soo Lee, Min Kim, So Young Park, Woo Sang Jung, Jin Wook Choi, Yong Cheol Lim, Ji Man Hong

**Affiliations:** 1 Department of Neurology, Ajou University School of Medicine, 164, World Cup-ro, Yeongtong-gu, Suwon-si, Gyeonggi-do 16499 South Korea; 2 Department of Radiology, Ajou University School of Medicine, Suwon, South Korea; 3 Department of Neurosurgery, Ajou University School of Medicine, Suwon, South Korea

**Keywords:** Neurology, Signs and symptoms, Neurological disorders, Stroke

## Abstract

Headache may represent acute phase of intracranial vertebrobasilar artery dissection (iVBAD). We aimed to evaluate its clinical significance in iVBAD. Consecutive acute iVBAD patients were grouped into ruptured iVBAD, unruptured iVBAD with no headache, isolated headache, or concurrent headache with neurological symptoms. Composite hemorrhagic/ischemic endpoints, and dynamic arterial changes were graded. Clinical characteristics of the four groups, and association between headache and composite outcomes was evaluated. Headaches were precedent in 79% of the ruptured iVBAD patients (maximal delay, 10D). In unruptured iVBAD, when patients with no headache (N = 69), concurrent headache (N = 111), and isolated headache (N = 126) were compared, concurrent headache was associated with ischemic endpoints (isolated headache as reference, adjusted odds ratio: 6.40, 95% confidence interval [2.03–20.19]). While there were no differences in hemorrhagic endpoints, dynamic arterial changes were higher in the isolated headache group (aOR: 3.98, 95% CI [1.72–9.18]) but not for the concurrent headache group (aOR: 1.59 [0.75–3.38]) compared to no headache group. Headache was more commonly severe (48.4% vs. 17.3%, p < 0.001) and ipsilateral (59.7% vs. 45.5%, p = 0.03) for isolated headache compared to concurrent headache, indicating a higher causal relationship. In iVBAD, isolated headache may be considered an acute-phase biomarker, associated with dynamic arterial changes.

## Introduction

Patients with intracranial vertebrobasilar artery dissection (iVBAD) may present with ruptured iVBAD resulting in subarachnoid hemorrhage (SAH), or with unruptured iVBAD, causing thrombotic events or headache^1^. Prediction of clinical outcomes for unruptured iVBAD is complicated because it needs to be evaluated multi-dimensionally. Outcomes may be classified as ischemic endpoints, such as functional dependence after ischemic stroke, early neurological deterioration (END), or subsequent ischemic stroke^2,3^. There may be hemorrhagic endpoints such as new SAH^3^ or arterial aneurysmal changes^4^ requiring acute or delayed intra-arterial embolization. Spontaneous arterial healing may also be an important outcome. While it is difficult to predict the timing of such complications, previous studies have reported that abrupt clinical alterations, such as rupture of dissecting aneurysms, occur in earlier phases^4^.

However, the diagnosis of iVBAD relies on angiographic images, which do not offer information regarding the temporal evolution of iVBAD. Headache identification may be important in this regard. Headaches may likely result from a direct tear of the blood vessel wall and thus occur in close temporal correlation with actual onset of arterial dissections^5,6^. Accordingly, it may be a biomarker for the acute phase of iVBAD, wherein dynamic clinical changes may more commonly occur. In contrast, for focal neurological deficits, this temporal relationship may be weaker as thrombotic events can occur later and be influenced by other systemic factors^7^.

The ICHD3 classifies the “headache associated with intracranial arterial dissections” and proposes factors that may be considered evidence of a causal relationship between headache and dissection such as severe intensity, sudden onset, and ipsilateral location^5^. However, a large number of iVBAD patients present with only neurological symptoms, without headaches. Furthermore, in patients with headaches, the patient’s headache may be isolated and be the chief reason for the patient to seek medical attention, or it may occur in concurrence with other neurological symptoms such as focal neurological deficits or vertigo. Whether such differences influence the clinical outcomes of iVBAD remains unknown.

## Aims and hypothesis

In this study, we hypothesized that the presence of headaches in patients with iVBAD may mark the acute phase of iVBAD, wherein rapid clinical and vascular changes may occur and, in turn, be associated with differences in outcomes. To confirm this hypothesis, we compared the clinical characteristics and outcomes according to presence of headache in a consecutive single-center database of patients with iVBAD who presented to the emergency department because of acute symptoms.

## Methods

### Study population and management

The institutional database for cervicocephalic arterial dissection was collected from retrospective medical records, as previously reported^7^. Patients who met the following criteria were included for analysis: (1) patients who presented between 2002 and 2021, with dissection nidus located at the intracranial vertebrobasilar arteries; and (2) patients who presented to the emergency department within 31 days from symptom onset to presentation.

### Classification of headache

The patients’ chief complaint, presence of headache, time from symptoms/headache onset to presentation, and headache characteristics were collected through a review of medical records. Based on these data, the patients were classified into four groups according to the accompanying headache. The first group comprised patients who presented with a ruptured iVBAD. Among patients with an unruptured iVBAD, those who presented with neurological symptoms or deficits without any headache were considered to have no headache. Patients with unruptured iVBAD who presented with isolated headaches which was the sole reason for seeking medical attention were classified as having isolated headaches. Patients with unruptured iVBAD presenting with both neurological symptoms and headache were classified into the concurrent headache group.

### Clinical and imaging variables

The arterial luminal morphology of the dissecting segment was described as steno-occlusive patterns and dilatation patterns (including stenosis and dilatation)^8^. The location of the dissection was categorized according to involvement of the basilar artery or vertebral artery only. If the patient presented with SAH, IA embolization via endovascular coiling was nearly always performed. In patients presenting with unruptured iVBAD, preemptive IA embolization via endovascular coiling/stent-assisted coiling or flow diverter stent insertion^9^ was selectively performed at the discretion of the attending physician in patients with fusiform/aneurysmal dilatation of the VAD with a diameter ratio between the dissecting and normal segments of the vertebral artery of ≥ 1.5 or progression of the dissection on follow-up images^10^. If it was performed during primary admission, it was classified as acute IA embolization, while if the patient was readmitted after discharge and embolization was performed in the later time frame, it was classified as delayed IA embolization. Antithrombotics including antiplatelets and anticoagulants were used at the discretion of the attending physician.

In patients with ischemic stroke, the initial clinical severity was graded using the National Institute of Health Stroke Scale (NIHSS), measured three times daily during acute stroke unit care and then once daily until discharge. END was classified as an increase in the NIHSS score by 2 or more points within 7 days post-admission^11^. Functional outcomes were graded at 3 months using the modified Rankin Scale (mRS) score. New ischemic stroke or SAH events that occurred during or after hospital admission were also identified.

Serial non-invasive angiographic images of the same modality (usually computed tomography angiographic images) were analyzed to evaluate spontaneous arterial healing in patients not treated with acute IA embolic treatment. Spontaneous arterial healing was classified as any improvement in the luminal diameter for stenotic or occlusive lesions and any decrease in the aneurysm size for dilatation patterns^8^. Aneurysmal changes or an increase in aneurysm size were specifically evaluated using the same imaging modalities.

### Definition of composite hemorrhagic endpoints, ischemic endpoints, and dynamic arterial changes

**Figure 1:**
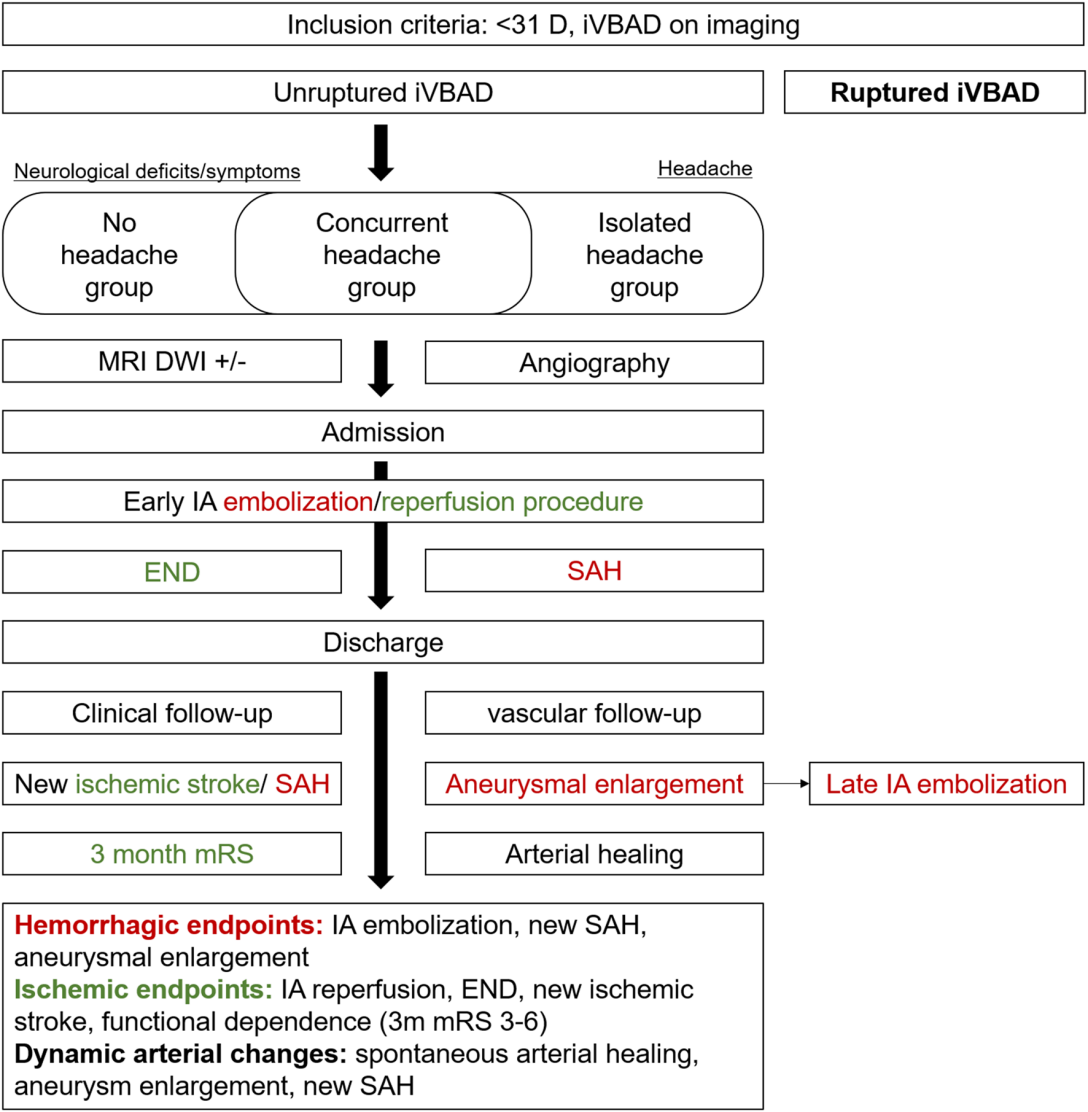
Study flowchart and classification of hemorrhagic, ischemic endpoints and dynamic arterial changes. *iVBAD* intracranial vertebrobasilar artery dissection, *MRI* magnetic resonance imaging, *DWI* diffusion weighted imaging, *IA* intra-arterial, *END* early neurological deterioration, *SAH* subarachnoid hemorrhage, *mRS* modified Rankin Scale.

Due to the heterogeneous potential outcome parameters in patients with unruptured iVBAD, these parameters were combined to form composite hemorrhagic and ischemic endpoints. Changes in arterial morphology were classified as dynamic arterial changes. For hemorrhagic endpoints, a composite of acute or delayed IA embolization therapy, new SAH, and aneurysmal enlargement was included. For ischemic endpoints, a composite of IA reperfusion therapy, END, functional dependence (3m mRS 3–6), and new ischemic stroke was included. For dynamic arterial changes, a composite of spontaneous arterial healing, aneurysm enlargement, and new SAH was included. A flowchart of classification of composite endpoints are shown in Fig. 1.

### Statistical analysis

First, a comparative analysis of baseline characteristics and temporal headache profiles was performed between the ruptured iVBAD, unruptured iVBAD presenting with isolated headache, concurrent headache, and no headache groups. Second, differences in hemorrhagic or ischemic endpoints or dynamic arterial changes were compared among the three unruptured iVBAD groups. A multiple logistic regression analysis was performed to confirm this relationship, consistently including trichotomized headache characteristics, age, and dissection morphology along with other significant variables identified in univariate analysis (p < 0.1). Third, the headache characteristics were compared between the isolated headache and concurrent headache groups. Continuous variables were compared using the Student’s *t*-test, Kruskal–Wallis test, or analysis of variance (ANOVA) test with Bonferroni post hoc tests. Categorical variables were analyzed using the chi-squared test or Fisher’s exact test. All statistical analyses were performed using IBM SPSS version 25.0, for Windows (IBM Corp., Armonk, NY, USA). Statistical significance was set at p < 0.05.

### Ethical approval and consent to participate

Ethics approval was obtained from the Ajou University Hospital Institutional Review Board (AJOUIRB-MDB-2021-674), and the study was performed following the ethical standards laid down in the 1964 Declaration of Helsinki and its later amendments. The Ajou University Hospital Institutional Review Board waived the need for obtaining patient consent.

## Results

### Clinical and imaging characteristics

Among the 371 iVBAD patients, 69 (18.6%) presented with no headache, 111 (29.9%) presented with concurrent headache, 126 (34.0%) presented with isolated headache, and 65 (17.5%) presented with a ruptured iVBAD (all comparisons performed in this order hereafter) (Table 1). While patients with a ruptured iVBAD were older (49 ± 11 vs. 47 ± 9 vs. 48 ± 11 vs. 52 ± 12, p = 0.033), there were more males (78.3% vs. 72.1% vs. 50.8% vs. 58.5%, p < 0.001) in the no headache group and concurrent headache groups. In terms of morphology, steno-occlusions were more common in the no headache and concurrent headache groups (68.1% vs. 64.0% vs. 26.2% vs. 1.5%), whereas dilatation patterns were more common in the isolated headache and ruptured iVBAD groups (31.9% vs. 36.0% vs. 73.8% vs. 98.5%, p < 0.001). Concurrent vascular risk factors such as hypertension (37.7% vs. 39.6% vs. 21.4% vs. 38.5%, p = 0.010) and diabetes mellitus (14.5% vs. 9.0% vs. 4.0% vs. 13.8%, p = 0.043) were lower in the isolated headache group, whereas dyslipidemia was highest in the no headache group, followed by the concurrent headache, isolated headache, and ruptured iVBAD groups (24.6% vs. 13.5% vs. 9.5% vs. 1.5%, p < 0.001).

**Table 1:** Comparison of clinical and imaging characteristics between the four groups.

	Unruptured iVBAD (N = 306)			Ruptured iVBAD (N = 65)	p-value
	No headache (N = 69)	Concurrent headache (N = 111)	Isolated headache (N = 126)		
Age	49 ± 11	48 ± 11	47 ± 9	52 ± 12	0.033*
Sex, male	54 (78.3%)	80 (72.1%)	64 (50.8%)	38 (58.5%)	< 0.001
Morphology					< 0.001
Steno-occlusion	47 (68.1%)	71 (64.0%)	33 (26.2%)	1 (1.5%)	
Dilatation	22 (31.9%)	40 (36.0%)	93 (73.8%)	64 (98.5%)	
Dissection location					0.054
VA	58 (84.1%)	89 (80.2%)	115 (91.3%)	59 (90.8%)	
BA involvement	11 (15.9%)	22 (19.8%)	11 (8.7%)	6 (9.2%)	
HTN	26 (37.7%)	44 (39.6%)	27 (21.4%)	25 (38.5%)	0.010
DM	10 (14.5%)	10 (9.0%)	5 (4.0%)	9 (13.8%)	0.043
Smoking	26 (37.7%)	37 (33.3%)	32 (25.4%)	13 (20.0%)	0.076
Dyslipidemia	17 (24.6%)	15 (13.5%)	12 (9.5%)	1 (1.5%)	< 0.001
HA	0 (0.0%)	111 (100.0%)	126 (100.0%)	49 (79.0%)	< 0.001
HA to presentation, d	–	3.0 [0.0–7.0]	7.0 [4.0–14.0]	0.0 [0.0–1.5]	< 0.001^†^
Maximal delay from headache, d	–	60	30	10	

*Ruptured iVBAD vs. isolated headache, p = 0.033.

^†^Isolated headache vs. concurrent headache, p = 0.002; isolated headache vs. ruptured iVBAD, p < 0.001; concurrent headache vs. ruptured iVBAD, p = 0.003, post hoc Bonferroni test.

*iVBAD* intracranial vertebrobasilar artery dissection, *VA* vertebral artery, *BA* basilar artery, *HTN* hypertension, *DM* diabetes mellitus.

Concomitant or preceding headache occurred in 79% of patients with ruptured iVBAD. The duration from headache onset to presentation was shorter than that in the concurrent headache or isolated headache groups (3.0 [0.0–7.0] D vs. 7.0 [4.0–14.0] D vs. 0.0 [0.0–1.5] D, p < 0.001), with a maximal delay from headache onset to presentation of 10 D.

### Hemorrhagic and ischemic endpoints

Patients who presented with unruptured iVBAD (N = 304) were further compared for multidimensional outcomes (Table 2). When patients with no headache (22.5%), concurrent headache (36.3%), and isolated headache (41.2%) were compared, there were significant differences in DWI lesions (75.4% vs. 82.0% vs. 2.4%, p < 0.001) as expected. The presence of headache in a trichotomized fashion was not associated with hemorrhagic endpoints (19.3% vs. 17.0% vs. 24.0%, p = 0.460) (Table 3A, multivariable analysis). Apart from the rates of delayed IA embolization therapy, which were higher in the isolated headache group (0.0% vs. 0.0% vs. 4.0%, p = 0.013), there were no differences in the rates of acute IA embolization therapy (13.0% vs. 10.8% vs. 12.7%, p = 0.872), aneurysmal enlargement (6.8% vs. 6.4% vs. 10.7%, p = 0.494), and new SAH (0.0% vs. 0.9% vs. 1.7%, p = 0.551).

**Table 2:** Comparison of multidimensional outcomes according to presence of headache in a trichotomized fashion in patients with unruptured iVBAD.

	No headache (N = 69)	Concurrent headache (N = 111)	Isolated headache (N = 126)	p-value
DWI lesion	52 (75.4%)	91 (82.0%)	3 (2.4%)	< 0.001
NIHSS	2.0 [1.0–4.0]	3.0 [1.0–4.0]	0.0 [0.0–0.0]	0.487
Hemorrhagic endpoints	11/57 (19.3%)	16/94 (17.0%)	25/104 (24.0%)	0.460
Acute IA embolization	9/69 (13.0%)	12/111 (10.8%)	16/126 (12.7%)	0.872
Delayed IA embolization	0/69 (0.0%)	0/111 (0.0%)	6/126 (4.8%)	0.013
Aneurysmal enlargement	4/59 (6.8%))	6/94 (6.4%)	11/103 (10.7%)	0.494
New SAH	0/64 (0.0%)	1/107 (0.9%)	2/117 (1.7%)	0.551
Ischemic endpoints	10/69 (14.5%)	27/111 (24.3%)	4/126 (3.2%)	< 0.001
IA reperfusion	1/69 (1.4%)	3/111 (2.7%)	0/126 (0.0%)	0.187
END	5/52 (9.6%)	14/91 (15.4%)	0/3 (0.0%)	0.489
3m mRS 3–6	5/66 (7.6%)	13 (11.9%)	3 (2.6%)	0.027
New ischemic stroke	3/64 (4.7%)	7/107 (6.5%)	1/117 (0.9%)	0.078
Dynamic arterial changes	25/52 (48.1%)	52/85 (61.2%)	76/93 (81.7%)	< 0.001
Arterial healing	22/50 (44.0%)	45/82 (54.9%)	64/89 (71.9)	0.003
Aneurysmal enlargement	4/59 (6.8%)	6/94 (6.4%)	11/103 (10.7%)	0.494
New SAH	0/64 (0.0%)	1/107 (0.9%)	2/117 (1.7%)	0.551

*DWI* diffusion-weighted imaging, *NIHSS* National Institute of Health Stroke Scale, *IA* intra-arterial, *END* early neurological deterioration, *mRS* modified Rankin Scale, *SAH* subarachnoid hemorrhage.

**Table 3:** Multiple logistic regression analysis showing association between presence of headache and various composite endpoints.

	Odd ratio [95% CI]	p-value
A. Hemorrhagic endpoints		
Presence of headache		0.717
No headache	Reference	
Isolated headache	0.70 [0.29–1.68]	0.42
Concurrent headache	0.83 [0.33–2.08]	0.069
Age	1.00 [0.97–1.04]	0.69
Morphology		
Steno-occlusion	Reference	
Dilatation	8.00 [3.45–18.52]	< 0.001
B. Ischemic endpoints		
Presence of headache		0.013
Isolated headache	Reference	
No headache	3.32 [0.91–12.06]	0.069
Concurrent headache	6.40 [2.03–20.19]	0.002
Age	1.04 [1.01–1.08]	0.03
Morphology		
Steno-occlusion	Reference	
Dilatation	1.93 [0.25–1.32]	0.19
Lesion location		
VA	Reference	
BA involvement	7.57 [3.24–17.70]	< 0.001
C. Dynamic arterial changes		
Presence of headache		0.004
No headache	Reference	
Isolated headache	3.98 [1.72–9.18]	0.001
Concurrent headache	1.59 [0.75–3.38]	0.226
Age	0.94 [0.91–0.97]	< 0.001
Morphology		
Steno-occlusion	Reference	
Dilatation	1.19 [0.60–2.27]	
HTN	0.49 [0.26–0.93]	0.029

*VA* vertebral artery, *BA* basilar artery, *HTN* hypertension.

The rates of ischemic endpoints were highest in the concurrent headache group, followed by the no headache and isolated headache groups (14.5% vs. 24.3% vs. 3.2%, p < 0.001) (Table 2). This difference was led by a higher rate of functional dependence (7.6% vs. 11.9% vs. 2.6%, p < 0.001), while differences in the rates of IA reperfusion (1.4% vs. 2.7% vs. 0.0%, p = 0.187), END (9.6% vs. 15.4% vs. 0.0%, p = 0.489), and new ischemic stroke (4.7% vs. 6.5% vs. 0.9%, p = 0.078) failed to reach statistical significance in its own. In multivariable analysis (Table 3B), concurrent headache was associated with a higher rate of ischemic endpoints than isolated headache (aOR: 6.40, 95% confidence interval [2.03–20.19], p = 0.002), which was expected. However, the difference between the concurrent headache and no headache groups was not statistically significant (aOR, 1.93; 95% CI [0.81–4.92], p = 0.14).

### Events of subarachnoid hemorrhage in unruptured iVBAD

**Figure 2:**
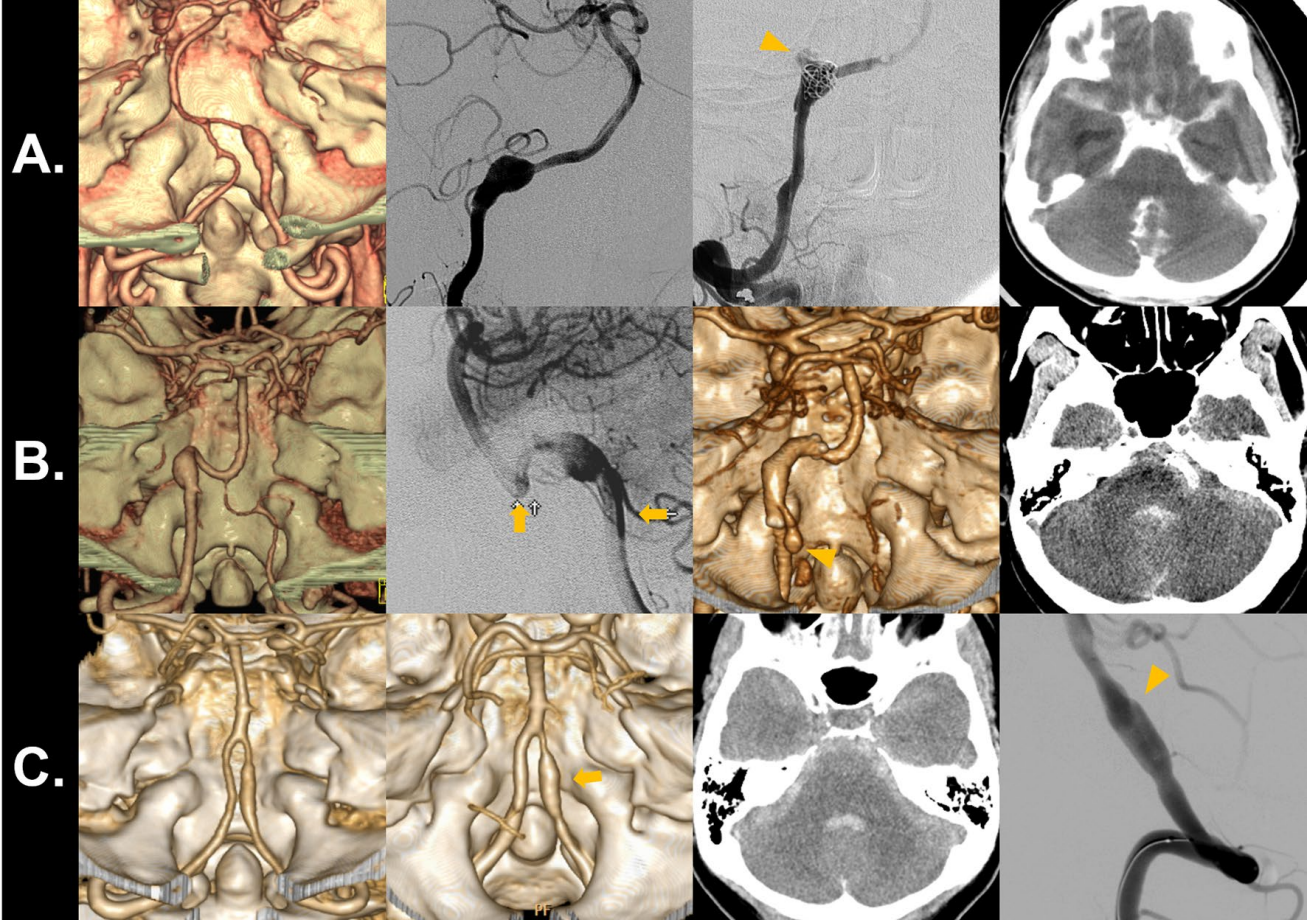
Imaging findings of events of subarachnoid hemorrhage in unruptured iVBAD. *iVBAD* intracranial vertebrobasilar artery dissection.

One new SAH event occurred in the concurrent headache group. A woman in her thirties presented with symptoms of lateral medullary infarction (Fig. 2A). CT angiography showed a dissecting aneurysm of the right V4 segment. Immediate IA embolization was planned. However, during embolization, initial guidewire advancement across the dissecting aneurysm resulted in contrast-extravasation (orange arrowhead) and intraprocedural subarachnoid hemorrhage.

Two new SAH event occurred in the isolated headache group. A man in his forties presented with headache and showed a dissecting aneurysm of his left vertebral artery (Fig. 2B). A Neuroform stent (Stryker Neurovascular, Kalamazoo, Michigan) was placed for flow diversion and as a pretreatment prior to coil embolization (orange arrows). It did not result in reduction of aneurysmal size. However, the patient did not agree to further embolization procedures. After 8 years, the patient presented with sudden mental change and SAH bleeding from the dissecting aneurysm (orange arrowhead). Another woman in her fifties presented with headache and dissecting aneurysm of the right vertebral artery (Fig. 2C). She was admitted for observation of the dissecting aneurysm. However, the patient developed sudden headache and coma on the next day. Brain imaging revealed SAH, and a slight enlargement (orange arrow) and lobulated appearance (orange arrowhead) of the aneurysm.

### Dynamic arterial changes

The rates of dynamic arterial changes were highest in the isolated headache group, followed by the concurrent headache and no headache groups (48.1% vs. 61.2% vs. 81.7%, p < 0.001). The most significant differences were observed in the rates of spontaneous arterial healing (44.0% vs. 54.9% vs. 71.9%, p = 0.003), while differences in aneurysmal enlargement (6.8% vs. 6.4% vs. 10.7%, p = 0.494) and new SAH (0.0% vs. 0.9% vs. 1.7%, p = 0.551) were not observed (Table 2). In the multivariable analysis (Table 3C), there was a positive association between dynamic arterial changes and isolated headache (aOR: 3.98, 95% CI [1.72–9.18], p = 0.001), while failing to reach statistical significance for concurrent headache (aOR: 1.59, 95% CI [0.75–3.38], p = 0.226) in comparison to no headache group.

### Comparison of characteristics of headache between isolated headache and concurrent headache groups

When headache features were compared between the concurrent headache and isolated headache groups (Table 4), the time from headache onset to presentation was shorter in the concurrent headache group (3.0 [0.0–7.0] D vs. 7.0 [4.0–14.0] D, p = 0.002). However, the rates of severe headache (17.8% vs. 48.4%, p < 0.001) and rates of headache location ipsilateral to dissection (45.5% vs. 59.7%, p = 0.030), rather than a diffuse pattern, were higher in the isolated headache group. These factors comprise the ICHD3 diagnostic criteria for “headache associated with intracranial arterial dissections,” and may be considered as evidence for a causal relationship between dissection and headache. There were no differences in the sudden onset of headaches in contrast to a gradual onset (43.6% vs. 36.3%, p = 0.252) of headaches.

**Table 4:** Comparison characteristics of headache in iVBAD.

	Concurrent headache (N = 111)	Isolated headache (N = 126)	p-value
HA to presentation, D	3.0 [0.0–7.0]	7.0 [4.0–14.0]	0.002
Severe headache	19 (17.3%)	60 (48.4%)	< 0.001
Headache location			0.443
Head	80 (72.7%)	83 (66.9%)	
Face or neck	27 (24.5%)	34 (27.4%)	
Combined	3 (2.7%)	7 (5.6%)	
Ipsilateral	50 (45.5%)	74 (59.7%)	0.030
Sudden onset	48 (43.6%)	45 (36.3%)	0.252
Onset pattern			
Preceded	58 (53.7%)		
Simultaneous	50 (46.3%)		

*iVBAD* intracranial vertebrobasilar artery dissection.

## Discussion

The study results show that in patients with iVBAD, most aneurysmal ruptures resulting in SAH occur within a short time interval from headache onset, which may be a temporal indicator of arterial wall tear. In acute unruptured iVBAD, patients presenting with isolated headache comprise a specific group of patients with a higher rate of arterial dilatation, isolated VA dissection, and fewer vascular risk factors. A lower rate of ischemic endpoints was observed in this specific group of patients along with higher rates of dynamic arterial changes. The clinical characteristics of the concurrent headache group resembled those of patients without headaches, and the rates of differences in dynamic arterial changes were not statistically significant. Headache characteristics were more homogenous in the isolated headache group than in the concurrent headache group, indicating a stronger causal relationship between headaches and iVBAD in the isolated headache group. However, under contemporary management for unruptured iVBAD, the presence of headaches did not seem to represent differences in hemorrhagic endpoints.

The short duration from headache onset to aneurysmal rupture and the higher frequency of dynamic arterial changes in the isolated headache group show that acute onset of headaches in iVBAD may indeed represent the early phase of arterial tear^6^ and its evolution. This association is clinically relevant because although the confirmation of an iVBAD relies on angiographic findings, it cannot represent the temporal profile of the dissections. Identification of the acute phase of iVBAD is clinically significant, as clinicians believe that dynamic clinical changes will occur in the acute phase. Mizutani also reported similar findings showing that most intracranial arterial dissections bleed within a few days of occurrence, when headache was used as a marker of its onset^4^. Propagation of arterial flaps or morphological changes will also theoretically occur in the acute phase of dissections, while arterial healing is usually thought to occur within 6 months of onset, and aggravation within 1 month^12^. A higher rate of dynamic arterial changes with the presence of headache, as shown in our study, point to this association. In contrast to headache, a proportion of ischemic events associated with dissections may occur at a time distant from arterial wall tear, and factors affecting systemic thrombosis, such as hypertension^13^ or pulse wave velocity^7^, may take part.

Our study results further reveal for the first time that not all headaches are equal in iVBAD. The association between dynamic arterial changes and headache is diluted in the concurrent headache group, while the time from headache onset to presentation was even shorter in this group. We believe that the heterogeneous headache mechanisms may play a role in concurrent headache. One such example is ‘headache attributed to ischemic stroke’ as defined by the ICHD3, which develops in very close relation to the clinical signs of ischemic stroke and can be of moderate intensity without specific characteristics, and either bilateral or unilateral^5^. It can occur in up to 6–44% of the ischemic stroke population, and posterior circulation stroke have greater odds of occurrence^14^. It may also occur before the onset of focal deficits, as a sentinel headache^15^, similar to headaches associated with dissections. Thus, in a significant proportion of the concurrent headache population, the onset of headache may mark the onset of an ischemic stroke, rather than a mechanical tear of the arterial wall. This difference can be also explained by the heterogeneity of concurrent headache and no headache groups, for they can be a mixture of acute ischemic VBAD and chronic stage VBAD concomitant with infarction due to pathologies such as atherosclerosis.

This study further broadens our understanding of the gray zone lying between the two extremes of clinical presentation of iVBAD, arterial rupture and thrombosis. The isolated headache group seems to represent a specific group of iVBAD, wherein mechanical tears of the arterial wall usually extend to the sub adventitial layer^16^, resulting in aneurysmal enlargement, but with favorable vascular profiles preventing acute rupture of the dissecting aneurysm. Younger age and favorable atherosclerotic vascular risk profiles^17^ of the isolated headache group compared to those of the ruptured iVBAD group shown in this study support this view. Morphologic features associated with iVBAD rupture such as significant proximal and distal stenosis, posterior inferior cerebellar artery involvement^18^, irregular surface, or stagnation sign^19^ have been identified, but it is limited by its retrospective nature, as arterial morphology may be influenced by the rupture event. Instead, there may be a higher risk of arterial rupture for sub adventitial iVBAD in patients with unfavorable vascular risk factor profiles. This is also supported by risk factors for cerebral aneurysm rupture^20–22^, such as smoking, alcohol, and hypertension, which at least partly overlap with atherosclerotic vascular risk factors. This may result in the natural selection of patients with favorable vascular profiles in the isolated headache group. A higher rate of arterial healing in this group can also be understood in this regard as vascular risk factors such as hyperlipidemia^23^, male sex, smoking^10^ and decreased endothelial function^7^ have been reported to be associated with reduced rates of vascular healing.

In contrast to our hypothesis, we did not find clinically significant differences in ischemic or hemorrhagic outcomes according to the trichotomized presence of headaches in patients with unruptured iVBAD other than a lower rate of ischemic endpoints in the isolated headache group, which is expected. For hemorrhagic endpoints, the event rate of aneurysmal enlargement or delayed rupture was probably not high enough to reach statistical significance. This finding agrees with previous studies reporting low rates of hemorrhage in unruptured intracranial arterial dissections^4,24,25^. While statistically insignificant due to low event rates, subsequent SAH events in unruptured iVBAD is worth attention. A total of 3 events occurred. One case was an intraprocedural complication, while in one case, stent insertion did not result in sufficient flow diversion and reduction of the aneurysm. If flow diversion treatment is planned for unruptured iVBAD, there must be a high degree of metal coverage^26^. Also, complete obliteration of the aneurysm must be confirmed by serial angiography^27^. One case of rupture of the dissecting aneurysm occurred in the acute phase, while the patient was admitted for observation. Subsequent spontaneous rupture of unruptured intracranial arterial dissections is a rare event. There is also difficulty in its prediction, as endovascular treatment is preemptively performed based on the clinician’s decision. Patients with morphologic features associated with iVBAD rupture discussed above should perhaps be more closely followed than those who do not. We believe that duration from the onset of headache should be also taken into consideration, as well as its improvement or aggravation. For ischemic endpoints, there was a tendency for elevated risk in the concurrent headache group compared to the no headache group, but the difference was not statistically significant. Theoretically, in patients with iVBAD presenting with thrombosis, extension of dissection flaps in the acute phase resulting in involvement of the basilar artery or posterior inferior cerebellar artery^28^ would cause early neurological deterioration or recurrent stroke. However, this study failed to show such an association. Future studies with larger patient populations may be required to address differences in hemorrhagic or ischemic endpoints.

This study has some limitations. First, as this was a retrospective observational study, patients suspected with high risk for hemorrhagic or ischemic complications were treated in a preemptive fashion. Thus, the natural differences in hemorrhagic or ischemic endpoints according to headache may have been minimized. Second, due to the long inclusion period, the results may have been biased by changes in the physician’s decision for arterial embolization. Accordingly, while the generalizability of the current results will need to be confirmed in prospective cohorts, our study is based on one of the largest iVBAD cohorts, and is therefore, of clinical value. Third, there were significant differences in baseline characteristics according to headache characteristics, especially in the isolated headache group. We however believe that this finding extends our understanding of the wide spectrum of presentation of iVBAD, and have carefully reviewed the possible causes of this difference and their implications in the discussions above. Fourth, we used a novel classification of composite ischemic and hemorrhagic endpoints and dynamic arterial changes. Such a classification has not been reported before, and whether it can represent the overall clinical outcomes of iVBAD needs to be validated.

In conclusion, in patients with acute iVBAD, headache may be considered an acute phase biomarker associated with early rupture of dissecting aneurysms and dynamic arterial changes. This association is stronger with isolated headaches and weaker with concurrent headaches. Headache profiles showed that the causal relationship between dissection and headache may be weaker in concurrent headaches. Under contemporary management for unruptured iVBAD, the presence of headaches does not seem to represent differences in ischemic or hemorrhagic outcomes, other than a lower rate of ischemic endpoints in the isolated headache group. We believe that the current study results extend our understanding of the various clinical presentations of iVBAD, and will be basis for more timely treatment decisions.

## Data Availability

Data supporting the findings of this study are available from the corresponding author upon reasonable request.
